# Outcomes Among Patients Hospitalized With Non–COVID-19 Conditions Before and During the COVID-19 Pandemic in Alberta and Ontario, Canada

**DOI:** 10.1001/jamanetworkopen.2023.23035

**Published:** 2023-07-12

**Authors:** Finlay A. McAlister, Anna Chu, Feng Qiu, Yuan Dong, Sean van Diepen, Erik Youngson, Amy Y. X. Yu, Charles de Mestral, Heather J. Ross, Peter C. Austin, Douglas S. Lee, Sameer S. Kadri, Harindra C. Wijeysundera

**Affiliations:** 1 Division of General Internal Medicine, Faculty of Medicine and Dentistry, University of Alberta, Edmonton, Alberta, Canada; 2 Alberta Strategy for Patient Oriented Research Support Unit, Edmonton, Alberta, Canada; 3 ICES (formerly Institute for Clinical Evaluative Sciences), Toronto, Ontario, Canada; 4 Department of Physical Therapy, Faculty of Medicine, University of Toronto, Toronto, Ontario, Canada; 5 Provincial Research Data Services, Alberta Health Services, Alberta, Canada; 6 Department of Critical Care Medicine, Faculty of Medicine and Dentistry, University of Alberta, Edmonton, Alberta, Canada; 7 Division of Cardiology, Department of Medicine, Faculty of Medicine and Dentistry, University of Alberta, Edmonton, Alberta, Canada; 8 Department of Medicine (Neurology), University of Toronto, Sunnybrook Health Sciences Centre, Toronto, Ontario, Canada; 9 Department of Surgery, Unity Health Toronto, Toronto, Ontario, Canada; 10 Department of Medicine (Cardiology), University of Toronto, Toronto, Ontario, Canada; 11 Peter Munk Cardiac Center, Ted Rogers Centre for Heart Research, University Health Network, Toronto, Ontario, Canada; 12 Institute of Health Policy, Management and Evaluation, Dalla Lana School of Public Health, University of Toronto, Toronto, Ontario, Canada; 13 Critical Care Medicine Department, National Institutes of Health Clinical Center, Bethesda, Maryland; 14 Critical Care Medicine Branch, National Heart, Lung and Blood Institute, Bethesda, Maryland

## Abstract

**Importance:**

The association of inpatient COVID-19 caseloads with outcomes in patients hospitalized with non–COVID-19 conditions is unclear.

**Objective:**

To determine whether 30-day mortality and length of stay (LOS) for patients hospitalized with non–COVID-19 medical conditions differed (1) before and during the pandemic and (2) across COVID-19 caseloads.

**Design, Setting, and Participants:**

This retrospective cohort study compared patient hospitalizations between April 1, 2018, and September 30, 2019 (prepandemic), vs between April 1, 2020, and September 30, 2021 (during the pandemic), in 235 acute care hospitals in Alberta and Ontario, Canada. All adults hospitalized for heart failure (HF), chronic obstructive pulmonary disease (COPD) or asthma, urinary tract infection or urosepsis, acute coronary syndrome, or stroke were included.

**Exposure:**

The monthly surge index for each hospital from April 2020 through September 2021 was used as a measure of COVID-19 caseload relative to baseline bed capacity.

**Main Outcomes and Measures:**

The primary study outcome was 30-day all-cause mortality after hospital admission for the 5 selected conditions or COVID-19 as measured by hierarchical multivariable regression models. Length of stay was the secondary outcome.

**Results:**

Between April 2018 and September 2019, 132 240 patients (mean [SD] age, 71.8 [14.8] years; 61 493 female [46.5%] and 70 747 male [53.5%]) were hospitalized for the selected medical conditions as their most responsible diagnosis compared with 115 225 (mean [SD] age, 71.9 [14.7] years, 52 058 female [45.2%] and 63 167 male [54.8%]) between April 2020 and September 2021 (114 414 [99.3%] of whom had negative SARS-CoV-2 test results). Patients admitted during the pandemic with any of the selected conditions and concomitant SARS-CoV-2 infection exhibited a much longer LOS (mean [SD], 8.6 [7.1] days or a median of 6 days longer [range, 1-22 days]) and greater mortality (varying across diagnoses, but with a mean [SD] absolute increase at 30 days of 4.7% [3.1%]) than those without coinfection. Patients hospitalized with any of the selected conditions without concomitant SARS-CoV-2 infection had similar LOSs during the pandemic as before the pandemic, and only patients with HF (adjusted odds ratio [AOR], 1.16; 95% CI, 1.09-1.24) and COPD or asthma (AOR, 1.41; 95% CI, 1.30-1.53) had a higher risk-adjusted 30-day mortality during the pandemic. As hospitals experienced COVID-19 surges, LOS and risk-adjusted mortality remained stable for patients with the selected conditions but were higher in patients with COVID-19. Once capacity reached above the 99th percentile, patients’ 30-day mortality AOR was 1.80 (95% CI, 1.24-2.61) vs when the surge index was below the 75th percentile.

**Conclusions and Relevance:**

This cohort study found that during surges in COVID-19 caseloads, mortality rates were significantly higher only for hospitalized patients with COVID-19. However, most patients hospitalized with non–COVID-19 conditions and negative SARS-CoV-2 test results (except those with HF or with COPD or asthma) exhibited similar risk-adjusted outcomes during the pandemic as before the pandemic, even during COVID-19 caseload surges, suggesting resiliency in the event of regional or hospital-specific occupancy strains.

## Key Points

**Question:**

What were the outcomes of patients hospitalized with non–COVID-19 conditions, even with negative SARS-CoV-2 test results, during the pandemic vs before the pandemic?

**Findings:**

In this cohort study of 132 240 patients hospitalized with common medical conditions, only those with heart failure or with chronic obstructive pulmonary disease or asthma exhibited a significantly higher risk-adjusted 30-day mortality during the pandemic (16% and 41% higher, respectively). As COVID-19 caseloads increased, lengths of stay and risk-adjusted mortality were significantly higher for patients with COVID-19 but not for those with non–COVID-19 conditions.

**Meaning:**

The findings show that most patients hospitalized with non–COVID-19 conditions (except heart failure or chronic obstructive pulmonary disease or asthma) exhibited similar risk-adjusted outcomes during the pandemic, even during COVID-19 caseload surges, suggesting resilience in the face of hospital occupancy strains.

## Teaser

This cohort study determines whether 30-day mortality and length of stay differed in patients hospitalized for non–COVID-19 conditions before and during the pandemic.

## Introduction

Before the COVID-19 pandemic, 18 of 30 observational studies reported increased inpatient mortality during times of hospital capacity strain.^1^ More recently, several studies have shown that surges in inpatient COVID-19 caseloads during the pandemic were associated with poorer outcomes for hospitalized patients with SARS-CoV-2^2,3,4^; however, the association of those surges with outcomes for patients hospitalized with non–COVID-19 conditions remains unclear. Studies showcasing the associations of the pandemic with outcomes in patients with non–COVID-19 conditions have yielded mixed results and were limited by predominantly focusing on specific conditions (eg, acute coronary syndrome [ACS]), care settings (eg, intensive care unit [ICU]), or the first wave of the pandemic.^2,3,4^ Furthermore, surge strain was usually measured using pandemic case counts that were not weighted for illness severity, resource needs, and hospital-specific bed constraints.

To understand the public health outcomes of caseload strain on non–COVID-19 conditions during the pandemic, we leveraged data from acute care hospitals in the single-payer universal access health care system of Canada in which all interactions with the health care system are captured. We explored the association between 30-day outcomes and an established severity-weighted metric of caseload strain^2^ for patients hospitalized with any of the 5 most common non–COVID-19 medical conditions requiring hospitalization: heart failure (HF), chronic obstructive pulmonary disease (COPD) or asthma, urinary tract infection (UTI) or urosepsis, ACS, or stroke.

## Methods

### Ethics

This cohort study used population-level, deterministically linked, health administrative data that were deidentified and, thus, deemed to be exempt from the need for individual patient consent by the University of Alberta Health Ethics Research Board. In Ontario, data sets were linked using unique encoded identifiers. The analysis of deidentified Ontario data was authorized under section 45 of Ontario’s Personal Health Information Protection Act. As health data privacy laws preclude the sharing of health data across provincial boundaries, the Alberta analyses were conducted within the Alberta Strategy for Patient Oriented Research Data and Research Services team, and the Ontario analyses were performed at ICES (formerly Institute for Clinical Evaluative Sciences), Toronto. This study followed the Strengthening the Reporting of Observational Studies in Epidemiology (STROBE) reporting guideline.

### Design, Sources of Data, and Medical Conditions of Interest

We performed a retrospective cohort study of all adult (aged ≥18 years) inpatient hospitalizations for similar periods before and after the COVID-19 pandemic onset (April 1, 2018, to September 30, 2019, vs April 1, 2020, to September 30, 2021, to mitigate seasonal effects) in 2 Canadian provinces (Alberta and Ontario, representing 50.3% of the total Canadian population). We excluded obstetric or psychiatric hospitalizations. Only the first hospitalization was included for each patient, and patients transferred between hospitals had the total hospital stay summed and attributed to the hospital in the sequence with the longest length of stay (LOS). We excluded patients who left against medical advice or were discharged to a correctional facility.

Among inpatients admitted after April 1, 2020, those with COVID-19 were identified using the COVID-19–specific *International Classification of Diseases, 10th Revision* (*ICD-10*) diagnosis code U07.1, which has sensitivity of 98%, specificity of 99%, and a positive predictive value of 92% to 93% in US and Alberta data.^5,6^ We used the Discharge Abstract Database, which captures all acute care hospitalizations and records up to 25 *ICD-10*–coded diagnoses and up to 20 procedure codes, to define our cohort and to build comorbidity profiles for each patient. Mortality, both in and out of the hospital, was captured from the Registered Persons Database in each province. For our analyses, we chose 5 indicator conditions accounting for the most common nondiscretionary medical case mix diagnoses in Alberta and Ontario: HF, COPD or asthma, UTI, ACS, and acute stroke.

### Surge Index (Exposure Variable)

We used a previously published surge index,^2^ which captures both the quantitative and qualitative burden in each hospital-month due to COVID-19 caseload relative to baseline bed capacity. This severity-weighted index has demonstrated prognostic utility in the outcomes of patients with COVID-19.^2^ The monthly surge index was calculated for each hospital in Alberta and Ontario after April 1, 2020, using the following equation (where *n* is number of admissions for COVID-19):

Surge index (per hospital-month) = ([(*n* without ICU, noninvasive positive pressure ventilation, or mechanical ventilation) + 2 × (*n* with noninvasive positive pressure ventilation or ICU) + 5 × (*n* with mechanical ventilation)] × 10) / (pre–COVID-19 baseline bed capacity of medical and ICU beds).

To calculate the surge index, we interrogated the Discharge Abstract Database and included admissions for patients with COVID-19; to determine the number admitted to the ICU, we included those who were admitted to a special care unit at any point during their index hospitalization (not just at baseline), even if not receiving ventilation, and identified patients requiring invasive mechanical ventilation or noninvasive positive pressure ventilation using Canadian Classification of Health Interventions procedure codes 1.GZ.31.CA and 1.GZ.31.CB, respectively. In response to the pandemic, some hospitals added acute ward and ICU beds in nontraditional spaces, and any patients admitted to these surplus areas were included in the numerator for calculating surge index but not in the denominator.

We grouped the surge index into prespecified shrinking categories (<50th percentile, 50th-74th percentile, 75th-89th percentile, 90th-99th percentile, and >99th percentile) to capture effects at extremes after excluding 22 (of 168) Ontario hospitals that did not admit any patients with COVID-19 during the pandemic (all Alberta acute care hospitals admitted patients with COVID-19). We did not include prepandemic data in the surge index analyses since by definition the surge index for all hospital-months before the pandemic would have been 0.

### Outcomes

The primary study outcome was 30-day all-cause mortality after admission with the 5 selected conditions or COVID-19 as the most responsible diagnosis. Length of stay was the secondary outcome. We compared outcomes (1) before vs during the pandemic for each of the conditions (since we only included the first hospitalization for any patient, none of those included in the prepandemic period were included in the postpandemic cohort if admitted for the same condition) and (2) across the surge index categories defined above.

### Statistical Analysis

We conducted analyses for each of the 5 selected conditions and COVID-19 separately (ie, 6 groups defined by the most responsible diagnosis for their index hospitalization). For all analyses, the patient was the unit of the analysis, and although the value of the surge index changed over time for each hospital, it was fixed for each patient since it was based on the date of the patient’s first hospitalization. We accounted for the clustering of patients within the hospital by using hierarchical (or multilevel) multivariable regression models that incorporated hospital-specific random effects to estimate the association between 30-day mortality and (1) study period (prepandemic vs pandemic) and (2) COVID-19 caseload surge category. Covariables for risk adjustment included age, sex, coronary artery disease, HF, hypertension, peripheral artery disease, ventricular arrythmias, atrial fibrillation or flutter, cancer, chronic pulmonary disease, other lung disease, dementia, diabetes, liver disease, peptic ulcer disease, kidney disease, cerebrovascular disease, and Charlson Comorbidity Index score. Race and ethnicity are not collected in routine Canadian administrative health care data and, thus, not included in the study. We used previously validated *ICD-10* code–based case definitions for diagnoses from the index hospitalization and any hospitalizations in the 12 months prior to the index admission and weighted the Charlson Comorbidity Index score using Schneeweiss weights.^7,8,9^ Other nonclinical covariables included type of hospital (teaching vs nonteaching), patient residence at the time of admission (rural vs urban, long-term care [LTC] facility vs community dwelling), socioeconomic status quintiles (assessed using the Pampalon deprivation index in Alberta and the Ontario Marginalization Deprivation Scale in Ontario), number of nonelective hospitalizations in the prior 12 months, and number of emergency department (ED) visits in the prior 6 months. All variables were prespecified and selected on the basis of potential for confounding. Risk-adjusted odds ratios (ORs) for 30-day all-cause mortality were plotted for patients with the 5 selected conditions without concomitant SARS-CoV-2 infection and for those with COVID-19 as the most responsible diagnosis across surge index categories. As some cell numbers were small, we pooled the adjusted ORs (AORs) for each province using random-effects meta-analysis using package metafor in R, version 1.4.0.^10^ In a sensitivity analysis, rather than examining the AOR for each of the prespecified surge index categories, we fit a logistic regression model for 30-day mortality, treating surge index as a continuous variable, and used restricted cubic splines with 4 knots at the fifth, 35th, 65th, and 95th percentiles to model the association between surge index and outcome.^11^ For LOS risk adjustment, we repeated the analyses using hierarchical linear models and the same covariables.

All analyses were performed using SAS, version 9.4 (SAS Institute Inc), and R, version 4.0.2 (R Foundation for Statistical Computing) statistical software. We used 2-sided tests of significance with *P* < .05, and significance levels were not adjusted to account for multiple comparisons (readers should interpret the results accordingly, particularly with respect to the baseline characteristics tables).

## Results

**Figure 1.**
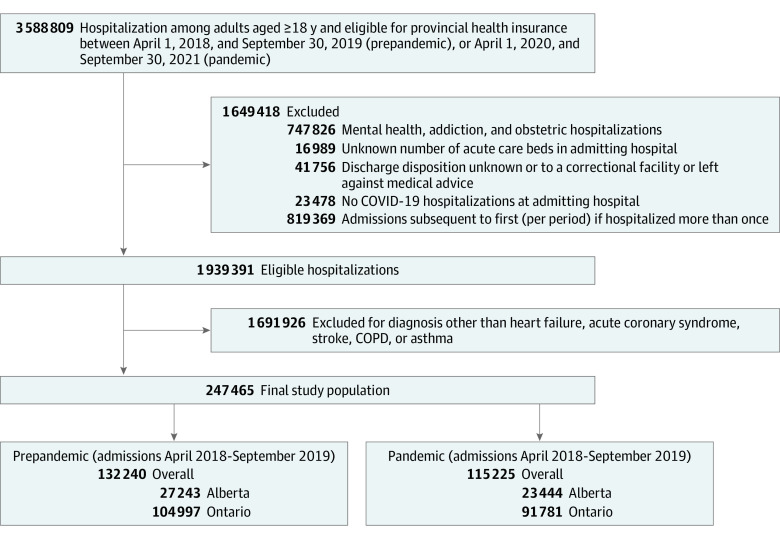
Cohort Creation Flowchart COPD indicates chronic obstructive pulmonary disease.

Between April 1, 2018, and September 30, 2019, 132 240 adults (mean [SD] age, 71.8 [14.8] years; 61 493 female [46.5%] and 70 747 male [53.5%]) were hospitalized for a most responsible diagnosis of any of the 5 indicator medical conditions in either province, compared with 115 225 (mean [SD] age, 71.9 [14.7] years; 52 058 female [45.2%] and 63 167 male [54.8%]) between April 1, 2020, and September 30, 2021 (114 414 [99.3%] of whom had negative SARS-CoV-2 test results) (Figure 1). The frequencies of the 5 selected medical conditions are listed in eTable 1 in Supplement 1. Between April 1, 2020, and September 30, 2021, 31 212 adults (mean [SD] age, 62.9 [17.2] years; 13 472 female [43.2%] and 17 740 male [56.8%]) were also admitted with a most responsible diagnosis of COVID-19 in the 2 provinces (Table 1).

**Table 1.** Characteristics of Study Population and Index Hospitalizations

Characteristic	Alberta				Ontario			
	Admitted for any of the 5 medical conditions, No. (%)		*P* value (pre- vs during pandemic)	Admitted for COVID-19, No. (%)^c^	Admitted for any of the 5 medical conditions, No. (%)		*P* value (pre- vs during pandemic)	Admitted for COVID-19, No. (%)^c^
	Prepandemic^a^	During pandemic^b^			Prepandemic^a^	During pandemic^b^		
No. of patients	27 243	23 444	NA	7409	104 997	91 781	NA	23 803
Age, mean (SD), y	70.26 (14.93)	70.69 (14.95)	.001	60.15 (17.37)	72.23 (14.59)	72.24 (14.41)	.82	63.77 (17.03)
Sex								
Female	12 290 (45.1)	10 370 (44.2)	.05	3151 (42.5)	49 203 (46.9)	41 688 (45.4)	<.001	10 321 (43.4)
Male	14 953 (54.9)	13 074 (55.8)	.08	4258 (57.5)	55 794 (53.1)	50 093 (54.6)	<.001	13 482 (56.6)
Rural residence	5037 (18.5)	4280 (18.3)	<.001	1325 (17.9)	14 174 (13.5)	12 263 (13.4)	.37	788 (3.3)
Deprivation quintile								
1 (least deprived)	3359 (12.3)	3105 (13.2)	<.001	880 (11.9)	17 458 (16.6)	15 995 (17.4)	<.001	3472 (14.6)
2	3666 (13.5)	3312 (14.1)		1083 (14.6)	19 041 (18.1)	16 901 (18.4)		4016 (16.9)
3	4411 (16.2)	3943 (16.8)		1156 (15.6)	20 150 (19.2)	17 647 (19.2)		4365 (18.3)
4	5462 (20.0)	4766 (20.3)		1404 (18.9)	21 938 (20.9)	18 837 (20.5)		5058 (21.2)
5 (most deprived)	6077 (22.3)	5217 (22.3)		2011 (27.1)	25 093 (23.9)	21 207 (23.1)		6701 (28.2)
Unknown	4268 (15.7)	3101 (13.2)		875 (11.8)	1317 (1.3)	1194 (1.3)		191 (0.8)
Long-term care resident	512 (1.9)	380 (1.6)	.03	128 (1.7)	4727 (4.5)	2688 (2.9)	<.001	1246 (5.2)
Nonelective hospitalization in the 12 mo prior to index hospitalization	3751 (13.8)	3067 (13.1)	.02	245 (3.3)	14 452 (13.8)	11 162 (12.2)	<.001	1217 (5.1)
Nonelective hospitalization in the 3 mo prior to index hospitalization	858 (3.1)	671 (2.9)	.06	17 (0.2)	3546 (3.4)	2510 (2.7)	<.001	363 (1.5)
Unplanned ED visit in the 6 mo prior to index hospitalization	3091 (11.3)	2390 (10.2)	<.001	100 (1.3)	35 775 (34.1)	26 778 (29.2)	<.001	8321 (35.0)
Comorbidities from index hospitalization episode and hospitalizations in prior year								
Coronary artery disease	9436 (34.6)	8440 (36.0)	.001	194 (2.6)	36 119 (34.4)	33 232 (36.2)	<.001	1257 (5.3)
Heart failure	7169 (26.3)	6646 (28.3)	<.001	231 (3.1)	30 115 (28.7)	27 757 (30.2)	<.001	1234 (5.2)
Hypertension	11 447 (42.0)	9679 (41.3)	.10	829 (11.2)	44 211 (42.1)	43 057 (46.9)	<.001	6939 (29.2)
Peripheral artery disease	404 (1.5)	254 (1.1)	<.001	26 (0.4)	1762 (1.7)	1726 (1.9)	.001	224 (0.9)
Ventricular arrythmias	357 (1.3)	315 (1.3)	.74	14 (0.2)	1575 (1.5)	1553 (1.7)	.001	82 (0.3)
Atrial fibrillation or flutter	4187 (15.4)	3078 (13.1)	<.001	332 (4.5)	17 271 (16.4)	15 990 (17.4)	<.001	1853 (7.8)
Cancer	900 (3.3)	643 (2.7)	<.001	117 (1.6)	4256 (4.1)	4083 (4.4)	<.001	787 (3.3)
COPD or asthma	8172 (30.0)	4959 (21.2)	<.001	559 (7.5)	27 989 (26.7)	17 112 (18.6)	<.001	1852 (7.8)
Dementia	1288 (4.7)	937 (4.0)	<.001	178 (2.4)	5626 (5.4)	4646 (5.1)	.003	1550 (6.5)
Diabetes	7834 (28.8)	7212 (30.8)	<.001	2270 (30.6)	32 572 (31.0)	29 017 (31.6)	.005	8096 (34.0)
Liver disease	342 (1.3)	247 (1.1)	.04	91 (1.2)	1052 (1.0)	1135 (1.2)	<.001	369 (1.6)
Other lung disease (non-COPD or asthma)	908 (3.3)	730 (3.1)	.16	167 (2.3)	2770 (2.6)	2416 (2.6)	.94	670 (2.8)
Peptic ulcer disease	187 (0.7)	187 (0.8)	.14	45 (0.6)	604 (0.6)	525 (0.6)	.92	134 (0.6)
Kidney disease	1480 (5.4)	1215 (5.2)	.21	221 (3.0)	5701 (5.4)	5059 (5.5)	.42	1012 (4.3)
Cerebrovascular disease	4515 (16.6)	4390 (18.7)	<.001	67 (0.9)	22 583 (21.5)	22 740 (24.8)	<.001	542 (2.3)
**Characteristics of index hospitalization**								
Schneeweiss Charlson Comorbidity Index score, mean (SD)	2.63 (1.97)	2.53 (1.92)	<.001	0.92 (1.43)	0.81 (1.61)	0.78 (1.56)	.001	0.34 (1.07)
No. of acute hospital beds								
<100	6019 (22.1)	4830 (20.6)	<.001	1689 (22.8)	18 899 (18.0)	16 431 (17.9)	<.001	2386 (10.0)
100-199	7984 (29.3)	6902 (29.4)		2541 (34.3)	23 898 (22.8)	21 306 (23.2)		3753 (15.8)
200-299	1387 (5.1)	1204 (5.1)		813 (11.0)	18 375 (17.5)	15 941 (17.4)		5855 (24.6)
300-399	4429 (16.3)	3742 (16.0)		1244 (16.8)	22 843 (21.8)	19 053 (20.8)		5142 (21.6)
400-499	3193 (11.7)	2995 (12.8)		624 (8.4)	6383 (6.1)	5624 (6.1)		2117 (8.9)
≥500	4231 (15.5)	3771 (16.1)		498 (6.7)	14 599 (13.9)	13 426 (14.6)		4550 (19.1)
Admission to teaching hospital	7889 (29.0)	7187 (30.7)	<.001	1243 (16.8)	30 811 (29.3)	27 475 (29.9)	.004	5483 (23.0)
Admission to ICU	1558 (5.7)	1662 (7.1)	<.001	1661 (22.4)	29 349 (28.0)	26 862 (29.3)	<.001	6340 (26.6)
Received mechanical ventilation	1345 (4.9)	1179 (5.0)	.64	1192 (16.1)	7540 (7.2)	6941 (7.6)	.001	3968 (16.7)
Total ICU time, median (IQR), h	78 (42-128)	53 (30-94)	<.001	211 (104-377)	61 (33-112)	60 (32-111)	.23	236 (110-508)
Episode length of stay, mean (SD), d	9.93 (20.89)	9.91 (18.10)	.91	11.88 (16.18)	8.85 (21.79)	8.88 (15.32)	.73	15.41 (24.68)
Discharge disposition								
Long-term care	1975 (7.2)	1747 (7.5)	<.001	335 (4.5)	23 599 (22.5)	17 261 (18.8)	<.001	2953 (12.4)
Home with home care	4334 (15.9)	3617 (15.4)		538 (7.3)	27 167 (25.9)	25 108 (27.4)		5015 (21.1)
Home without home care	19 219 (70.5)	16 379 (69.9)		5455 (73.6)	47 117 (44.9)	42 774 (46.6)		11 591 (48.7)
Death	1715 (6.3)	1701 (7.3)		1081 (14.6)	7114 (6.8)	6638 (7.2)		4244 (17.8)
Surge index, mean (SD)	NA^d^	2.81 (3.88)	NA	8.31 (7.14)	NA^d^	2.55 (3.90)	NA	8.89 (7.56)

Abbreviations: COPD, chronic obstructive pulmonary disease; ED, emergency department; ICU, intensive care unit; NA, not applicable.

^a^ April 1, 2018, to September 30, 2019.

^b^ April 1, 2020, to September 30, 2021.

^c^ As the most responsible diagnosis.

^d^ By definition, the COVID-19 caseload surge index was NA prepandemic since the number of cases was 0.

### Differences in Admitted Patients With Indicator Conditions Before vs After Pandemic Onset

Patient age, sex, and LOS were similar before and during the pandemic for hospitalizations for the 5 selected conditions (Table 1). However, patients hospitalized with any of the 5 conditions during the pandemic (vs before) were less likely to come from LTC facilities, exhibited fewer ED visits or hospitalizations in the prior 3 to 12 months, had lower comorbidity scores, and were less likely to have COPD, asthma, or dementia, but were more likely to have cardiac or cerebrovascular disease and to be admitted to teaching hospitals (Table 1).

### Differences in Admitted Patients During the Pandemic by COVID-19 Status

Compared with patients admitted with any of the 5 selected conditions during the pandemic, patients admitted with COVID-19 as their most responsible diagnosis were younger, more likely to be male and from urban areas or LTC facilities, had lower comorbidity burdens (particularly less COPD or asthma, cardiac disease, cerebrovascular disease, or chronic kidney disease), were less likely to have had recent hospitalizations, and had much longer LOS, greater need for ICU admission, and higher in-hospital mortality (Table 1).

### Differences in Admitted Patients With the 5 Selected Conditions Across COVID-19 Surge Categories During the Pandemic

Compared with patients admitted in low-surge periods, those admitted with the 5 indicator conditions when hospitals were surging with higher COVID-19 caseloads were younger, had lower comorbidity scores, and had fewer prior hospitalizations; however, patients who were admitted exhibited greater need for ICU admission and ventilatory support (eTable 2 in Supplement 1). We also observed an apparent dose-dependent association with triage of patients without COVID-19 based on COVID-19 caseloads. At higher surge index times, the proportion of admitted patients who were older, were from rural areas, had high comorbidity scores, or had prior hospitalizations were all significantly lower (eTable 2 in Supplement 1).

### Outcomes of Hospitalized Patients With the 5 Selected Conditions Before vs After Pandemic Onset

**Figure 2.**
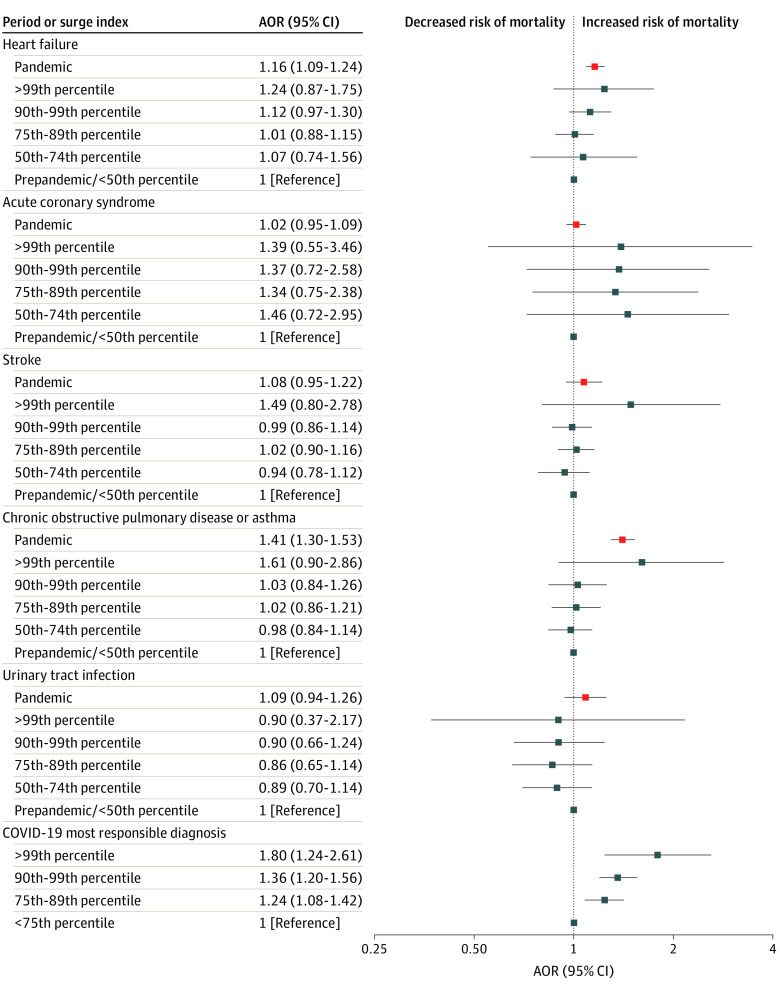
Adjusted Risk of 30-Day Mortality Red markers represent risk-adjusted 30-day mortality with 95% CIs during vs before the COVID-19 pandemic for each most responsible diagnosis, and the blue markers represent risk-adjusted 30-day mortality with 95% CIs across surge index categories during the pandemic for patients with each of the 5 selected conditions (and without SARS-CoV-2 infection) and for patients COVID-19 as the most responsible diagnosis. Note that the reference category for patient admissions for COVID-19 as the most responsible diagnosis was less than the 75th percentile rather than less than the 50th percentile due to small numbers in the less than 50th percentile group. Risk adjustment covariables are listed in the Statistical Analysis.

Despite their more favorable age and comorbidity profiles at baseline, the 115 225 patients hospitalized for any of the 5 indicator conditions during the pandemic exhibited poorer outcomes than the 132 240 admitted for the same diagnoses prepandemic, with more requiring ICU admission (28 524 [24.8%] vs 30 907 [23.4%]; *P* < .001) and more dying (8339 [7.2%] vs 8829 [6.7%]; *P* < .001), but this was largely driven by the markedly poorer outcomes in the 811 patients (0.7%) with concomitant SARS-CoV-2 infection (Table 2). In fact, patients admitted during the pandemic with any of the indicator conditions and concomitant SARS-CoV-2 infection exhibited much longer LOS (mean [SD], 8.6 [7.1] days or a median of 6 days longer [range, 1-22 days]) and greater mortality (varying from no increase in patients with stroke to a 10.5% absolute increase in patients with COPD or asthma, with an overall mean [SD] absolute increase at 30 days of 4.7% [3.1%]) than those without coinfection (all *P* < .001) (Table 2). Our analysis of outcomes for patients hospitalized with any of the 5 selected conditions without concurrent SARS-CoV-2 infection showed similar LOS before vs after March 2020 (Table 2), but patients with HF (AOR, 1.16; 95% CI, 1.09-1.24) and COPD or asthma (AOR, 1.41; 95% CI, 1.30-1.53) had higher risk-adjusted 30-day mortality during the pandemic than before (Figure 2).

**Table 2.** Outcomes Stratified by Surge Index

Condition and period or surge index category	Admitted with medical condition and tested negative for SARS-CoV-2 infection				Admitted with medical condition and tested positive for SARS-CoV-2 infection			
	No. of patients, both provinces combined	30-d mortality, both provinces combined, No. (%)	Alberta LOS, median (IQR), d	Ontario LOS, median (IQR), d	No. of patients, both provinces combined	30-d mortality, both provinces combined, No. (%)	Alberta LOS, median (IQR), d	Ontario LOS, median (IQR), d
**Heart failure**								
Prepandemic^a^	27 137	2542 (9.37)	7 (4-13)	6 (3-10)	NA	NA	NA	NA
Pandemic^b^	26 131	2726 (10.43)	7 (4-13)	6 (3-11)	186	25 (13.44)	29 (7-61)	18 (7-36)
Surge index percentile								
<50th	5474	613 (11.2)	7 (4-13)	6 (3-11)	≤5	0 (0)	Suppressed^c^	Suppressed^c^
50th-74th	9268	922 (9.95)	7 (4-13)	6 (3-11)	30	4 (13.33)	29 (16-69)	12 (7-32)
75th-89th	6253	635 (10.16)	7 (4-13)	6 (4-11)	54	6 (11.11)	19 (7-39)	21 (6-40)
90th-99th	4760	511 (10.74)	7 (4-13)	6 (4-11)	86	12 (13.95)	30 (4-61)	19 (8-34)
>99th	376	45 (11.97)	6 (4-12)	7 (4-11)	12	≤5	Suppressed^c^	10 (9-22)
**Acute coronary syndrome**								
Prepandemic^a^	35 497	2204 (6.21)	4 (3-7)	4 (2-7)	NA	NA	NA	NA
Pandemic^b^	33 191	2016 (6.07)	4 (3-6)	4 (2-7)	179	20 (11.17)	5 (4-14)	6 (3-19)
Surge index percentile								
<50th	6948	422 (6.07)	4 (3-7)	4 (3-7)	≤5	0 (0)	Suppressed^c^	Suppressed^c^
50th-74th	11504	721 (6.27)	4 (3-6)	4 (2-7)	32	≤5	Suppressed^c^	7 (3-30)
75th-89th	8123	483 (5.95)	4 (3-6)	4 (2-6)	41	≤5	Suppressed^c^	6 (3-13)
90th-99th	6153	365 (5.93)	4 (3-6)	3 (2-6)	90	13 (14.44)	6 (4-14)	6 (3-20)
>99th	463	25 (5.40)	4 (3-6)	3 (2-6)	13	≤5	Suppressed^c^	6 (3-12)
**Stroke**								
Prepandemic^a^	25 667	3431 (13.37)	5 (3-13)	6 (3-12)	NA	NA	NA	NA
Pandemic^b^	25 735	3458 (13.44)	5 (3-13)	6 (3-12)	280	41 (14.64)	16 (5-46)	26 (11-53)
Surge index percentile								
<50th	4673	634 (13.57)	6 (3-17)	6 (3-12)	6	≤5	NA	96 (33-314)
50th-74th	9347	1257 (13.45)	5 (3-13)	6 (3-13)	43	≤5	49 (14-92)	28 (8-72)
75th-89th	6666	896 (13.44)	5 (3-13)	6 (4-12)	84	12 (14.29)	22 (5-52)	33 (13-56)
90th-99th	4694	619 (13.19)	5 (3-12)	6 (4-13)	129	22 (17.05)	10 (4-21)	23 (10-48)
>99th	355	52 (14.65)	5 (2-9)	6 (3-11)	18	≤5	Suppressed^c^	14 (12-23)
**COPD or asthma**								
Prepandemic^a^	30 262	1961 (6.48)	5 (3-8)	4 (2-7)	NA	NA	NA	NA
Pandemic^b^	16 888	1461 (8.65)	5 (3-8)	4 (2-7)	68	13 (19.12)	11 (5-24)	10 (4-24)
Surge index percentile								
<50th	4574	419 (9.16)	5 (3-8)	4 (2-7)	≤5	0 (0)	Suppressed^c^	Suppressed^c^
50th-74th	6047	502 (8.3)	5 (3-8)	4 (2-7)	15	≤5	Suppressed^c^	5 (2-16)
75th-89th	3689	315 (8.54)	5 (3-9)	4 (2-7)	20	≤5	18 (7-27)	6 (3-26)
90th-99th	2386	204 (8.55)	5 (3-9)	4 (2-7)	30	6 (20.00	7 (5-13)	21 (7-38)
>99th	192	21 (10.94)	5 (2-7)	4 (2-8)	≤5	0 (0)	Suppressed^c^	Suppressed^c^
**Urinary tract infection**								
Prepandemic^a^	14 127	581 (4.11)	5 (3-9)	4 (2-7)	NA	NA	NA	NA
Pandemic^b^	12867	543 (4.22)	6 (3-10)	4 (3-8)	100	8 (8.00)	9 (5-27)	7 (4-45)
Surge index percentile								
<50th	3324	152 (4.57)	5 (3-10)	4 (3-8)	≤5	0 (0)	NA	Suppressed^c^
50th-74th	4679	191 (4.08)	6 (3-10)	4 (3-8)	23	≤5	10 (8-68)	5 (3-57)
75th-89th	2826	116 (4.1)	5 (3-10)	4 (3-8)	26	≤5	11 (5-87)	20 (7-51)
90th-99th	1887	78 (4.13)	5 (3-9)	4 (3-8)	42	≤5	6 (4-18)	7 (5-32)
>99th	151	6 (3.97)	7 (3-10)	5 (3-8)	≤5	≤5	Suppressed^c^	Suppressed^c^
**Any of the selected conditions**								
Prepandemic^a^	132 240	10 659 (8.06)	5 (3-9)	5 (3-9)	NA	NA	NA	NA
Pandemic^b^	114 414	10 148 (8.87)	5 (3-10)	5 (3-9)	811	107 (13.19)	12 (5-38)	15 (5-38)
Surge index percentile								
<50th	24 861	2228 (8.96)	5 (3-10)	5 (3-9)	20	≤5	Suppressed^c^	22 (4-150)
50th-74th	40 730	3580 (8.79)	5 (3-9)	5 (3-9)	143	17 (11.89)	26 (10-92)	11 (4-41)
75th-89th	27 475	2423 (8.82)	5 (3-10)	5 (3-9)	225	25 (11.11)	13 (5-45)	19 (5-40)
90th-99th	19 815	1768 (8.92)	5 (3-9)	5 (3-9)	375	57 (15.20	8 (4-24)	17 (6-36)
>99th	1533	149 (9.72)	4 (3-8)	5 (3-10)	48	7 (14.58)	Suppressed^c^	10 (6-17)

Abbreviations: COPD, chronic obstructive pulmonary disease; LOS, length of stay; NA, not applicable.

^a^ April 1, 2018, to September 30, 2019.

^b^ April 1, 2020, to September 30, 2021.

^c^ For privacy concerns, results and counts have been suppressed in cells where there are 5 or fewer individuals.

### COVID-19 Caseloads During the Pandemic and Outcomes of Hospitalized Patients With the 5 Selected Conditions

**Figure 3.**
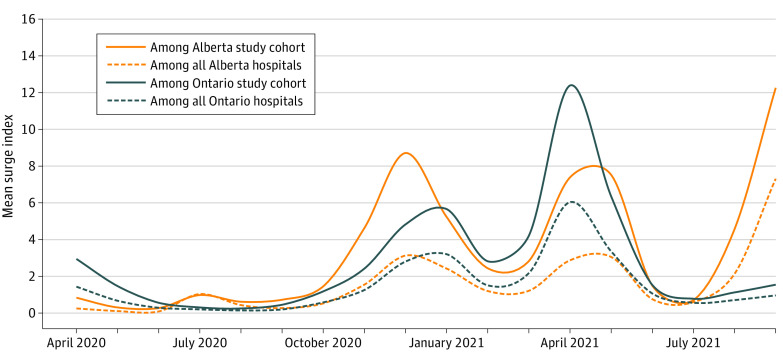
Average Hospital Surge Indices by Month in Each Province, April 2020 to September 2021 Among all Alberta or Ontario hospitals indicates the surge indices based on all nonobstetric, non–mental health, or addictions hospitalizations; among study cohort, the surge indices are calculated based only on patients admitted with any of the 5 selected conditions or COVID-19.

During the pandemic, the 146 Ontario hospitals that admitted patients with COVID-19 exhibited a mean monthly surge index of 1.49 (ranging from a low of 0.12 in August 2020 to a high of 6.03 in April 2021), and the 89 Alberta hospitals exhibited a mean surge index of 1.59 (ranging from a low of 0.07 in June 2020 to a high of 7.30 in September 2021) (Figure 3). Examination of outcomes across surge strata during the pandemic revealed that although hospitals became busier, risk-adjusted 30-day mortality and LOS did not exhibit statistically significant differences for patients with any of the 5 selected conditions without concomitant SARS-CoV-2 infection when data were pooled across both provinces (Figure 2; eTable 3 in Supplement 1). There was also no association between outcomes for the 5 indicator conditions when the surge index was analyzed as a continuous variable in our sensitivity analysis. Thirty-day mortality and LOS for patients with any of the 5 indicator conditions were also stable across pandemic waves (eFigure in Supplement 1). Although LOS was stable across surge categories for patients with COVID-19 (eTable 4 in Supplement 1), risk-adjusted 30-day mortality was significantly associated with the surge index for patients with COVID-19 (AOR, 1.80; 95% CI, 1.24-2.61 for the greater than 99th percentile group vs the less than 75th percentile group) (eTable 3 in Supplement 1; Figure 2). Of note, the reference surge index category for admissions with COVID-19 as the most responsible diagnosis was less than the 75th percentile rather than less than the 50th percentile due to small numbers per hospital in the less than 50th percentile group (cell numbers <6 are required to be suppressed due to the legal boundaries around the release and analysis of health information in each province).

## Discussion

In this cohort study of patients hospitalized in Alberta and Ontario before and during the COVID-19 pandemic, we found that despite having better chronic risk factor profiles, patients admitted during the pandemic for the 5 selected conditions (HF, COPD or asthma, UTI, ACS, or stroke) exhibited poorer outcomes (more required ICU admission and more died). These findings may be due to markedly worse outcomes in the 0.7% of patients with concomitant SARS-CoV-2 infection in our cohort. However, it is important to note that patients hospitalized with HF or with COPD or asthma and without SARS-CoV-2 infection still exhibited risk-adjusted 30-day mortality rates of 16% and 41% higher than those admitted before the pandemic. While these patients may have had more severe illness when admitted to the hospital than those with the same conditions admitted before the pandemic, we did adjust for most covariables known to be associated with mortality in HF or COPD (we did not have data on smoking, body mass index, ejection fraction, or Acute Physiology and Chronic Health Evaluation scores). Alternately, it is possible that patients with HF or COPD or asthma exhibited poorer outcomes during the pandemic as they were more directly competing for the same scarce mechanical ventilation resources as patients with COVID-19 compared with the other selected conditions. We do not believe that these patients with HF or COPD or asthma had undiagnosed COVID-19, as screening of all hospitalizations for SARS-CoV-2 infection became routine early in the pandemic in Alberta and Ontario.^12,13^ We believe it is not surprising that outcomes did not appreciably change for patients with ACS or stroke, since most hospitals have expedited management pathways for these conditions that were still in force even during the pandemic.

Our findings that patients hospitalized with a most responsible diagnosis of any of the 5 selected conditions during the pandemic were less likely to come from LTC facilities, had fewer prior ED visits or hospitalizations, and had lower comorbidity burdens suggest that LTC residents or patients with comorbidities may have been following public health advice to shelter in place and reduce their exposure risk to COVID-19. Certainly, LTC facilities instituted more stringent rules around hospital transfers and repatriation early in the pandemic in Canada.^13^ An alternative explanation may be a manifestation of collider bias in which disposition outcomes for LTC residents or patients with high comorbidity burdens who contracted COVID-19 may have been death prior to hospitalization or triaged in the ED to options other than acute care hospitalization.^14^ Regardless of the possible cause, this finding is important for public health approaches to future pandemics and for research studies of outcomes during the COVID-19 pandemic that do not include comprehensive adjustment for confounders beyond just age, sex, and Charlson Comorbidity Index scores.

Our finding that mortality risk for patients with COVID-19 was significantly associated with hospital caseloads (with higher 30-day death rates at times of surges) confirms data from other countries.^1,2,3,4^ However, we found that outcomes were relatively stable across surge index strata for patients hospitalized during the pandemic for non–COVID-19 medical conditions. This finding is contrary to the commonly held belief, even in a survey of hospital administrators, that inpatients without COVID-19 received poorer quality of care and experienced worse outcomes during COVID-19 surges.^15^ Our findings suggest that the Canadian health care system may have, for the most part, weathered the storm during the pandemic or at least may have been used to running at near capacity even before the pandemic. In Canada, the average hospital occupancy rate was 92% in 2019, the highest of all Organization for Economic Co-operation and Development (OECD) countries, which is not surprising since Canada ranks 33rd of 36 OECD countries in the number of acute care beds (at 2.5 per 1000 people vs the OECD average of 4.4 per 1000 people in 2019).^16^ By comparison, average acute care bed occupancy in the US was 64% before the pandemic.^16^ However, although 30-day mortality and LOS were not significantly associated with COVID-19 caseloads, there is no doubt that the diversion of staff and resources to patients with COVID-19 has resulted in a substantial accrued care deficit in Canada (and worldwide).^17^

### Limitations

Although we report all hospitalizations in 2 of Canada’s most populous provinces and were able to account for out-of-hospital deaths, there are some limitations to our study. First, as with any observational study, there is the possibility of bias due to unmeasured confounders, and we were unable to account for heterogeneity in management practices between hospitals and clinicians over the course of the pandemic, as well as the association of variants and vaccination.^18^ Second, we did not have data on do-not-resuscitate status, other predesignated treatment limitations, or in-hospital or outpatient medication use. We also did not have access to clinical variables such as oxygen saturation, blood pressure, heart rate, results of blood or radiology tests, and duration of symptoms at admission to fully adjust for disease severity of the most responsible diagnoses. Third, if some patients were not tested for SARS-CoV-2, we may have misclassified them as not having COVID-19, which would have biased our findings toward the null; however, by April 2020, screening for SARS-CoV-2 infection in all patients at the time of admission was routine in both provinces.^13^ Fourth, we relied on administrative rather than clinical data to define comorbidities, but we did use well-validated *ICD-10*–based case definition algorithms. Fifth, we only examined outcomes for hospitalized patients and, thus, may have underestimated the adverse outcomes on a population basis of COVID-19 caseload surges since at-home deaths were not accounted for. In a similar vein, since we only examined outcomes for first hospitalizations for any of the selected conditions, we may have underestimated mortality outcomes, because patients hospitalized 2 or more times for the same condition in a short period have poorer outcomes. Sixth, although there appears to be a large difference between Alberta and Ontario in ICU usage for patients with the 5 selected diagnoses or COVID-19, this appears to be merely a labeling issue in what was classified as an ICU in each province by the health authorities, since the proportions of patients receiving mechanical ventilation in both provinces were relatively similar. Seventh, it should be acknowledged that the surge index number cannot be used to extrapolate the percentage of available hospital resources devoted to the care of patients with COVID-19 at any one time; rather, it was used as a metric to stratify COVID-19 caseloads (by percentile) between hospitals and across phases of the pandemic.

## Conclusions

In this cohort study, hospital admissions for 5 most common most responsible diagnoses in Ontario and Alberta decreased during the first 18 months of the COVID-19 pandemic, mirroring data from other jurisdictions.^19,20,21^ While surges in COVID-19 caseloads were associated with significantly higher mortality rates for patients with COVID-19, most patients hospitalized with non–COVID-19 conditions and negative SARS-CoV-2 test results (except those with HF or with COPD or asthma) exhibited similar risk-adjusted outcomes (30-day mortality and LOS) during the pandemic, even during COVID-19 caseload surges. It is interesting to speculate to what extent these observations reflect the fact that the Canadian hospital system was used to running at near capacity even before the pandemic. Regardless, our results provide reassuring evidence of resilience in the event of future regional or hospital-specific occupancy strains (eg, mass casualty events, future pandemics).

## Notes

Supplement 1. **eTable 1.** Frequency of the Medical Indicator Conditions **eTable 2.** Baseline Characteristics of Patients Hospitalized During the COVID-19 Pandemic, by Surge Stratum **eTable 3.** Adjusted Risk of 30-Day Mortality and Length of Stay Among Patients Hospitalized During the COVID-19 Pandemic **eTable 4.** Outcomes for Patients Hospitalized With COVID-19 as a Most Responsible Diagnosis Between April 2020 and September 2021 **eFigure.** Crude Outcome Rates by Wave and Surge Index, Alberta and Ontario Combined

Supplement 2.
Nonauthor Collaborators

Supplement 3.
Data Sharing Statement

## References

[1] Eriksson CO, Stoner RC, Eden KB, Newgard CD, Guise JM. The association between hospital capacity strain and inpatient outcomes in highly developed countries: a systematic review. J Gen Intern Med. 2017;32(6):686-696. doi:10.1007/s11606-016-3936-3 27981468 PMC5442002

[2] Kadri SS, Sun J, Lawandi A, . Association between caseload surge and COVID-19 survival in 558 U.S. hospitals, March to August 2020. Ann Intern Med. 2021;174(9):1240-1251. doi:10.7326/M21-1213 34224257 PMC8276718

[3] Bottle A, Faitna P, Aylin PP. Patient-level and hospital-level variation and related time trends in COVID-19 case fatality rates during the first pandemic wave in England: multilevel modelling analysis of routine data. BMJ Qual Saf. 2022;31(3):211-220. doi:10.1136/bmjqs-2021-012990 34234008

[4] Bravata DM, Perkins AJ, Myers LJ, . Association of intensive care unit patient load and demand with mortality rates in US Department of Veterans Affairs hospitals during the COVID-19 pandemic. JAMA Netw Open. 2021;4(1):e2034266. doi:10.1001/jamanetworkopen.2020.34266 33464319 PMC7816100

[5] Kadri SS, Gundrum J, Warner S, . Uptake and accuracy of the diagnostic code for COVID-19 among US hospitalizations. JAMA. 2020;324(24):2553-2554. doi:10.1001/jama.2020.20323 33351033 PMC7756233

[6] Wu G, D’Souza AG, Quan H, . Validity of ICD-10 codes for COVID-19 patients with hospital admissions or ED visits in Canada: a retrospective cohort study. BMJ Open. 2022;12(1):e057838. doi:10.1136/bmjopen-2021-057838 35063962 PMC8787827

[7] McAlister FA, Dong Y, Chu A, ; CORONA Collaboration. The risk of death or unplanned readmission after discharge from a COVID-19 hospitalization in Alberta and Ontario. CMAJ. 2022;194(19):E666-E673. doi:10.1503/cmaj.220272 35577377 PMC9438727

[8] Quan H, Sundararajan V, Halfon P, . Coding algorithms for defining comorbidities in ICD-9-CM and ICD-10 administrative data. Med Care. 2005;43(11):1130-1139. doi:10.1097/01.mlr.0000182534.19832.83 16224307

[9] Schneeweiss S, Wang PS, Avorn J, Glynn RJ. Improved comorbidity adjustment for predicting mortality in Medicare populations. Health Serv Res. 2003;38(4):1103-1120. doi:10.1111/1475-6773.00165 12968819 PMC1360935

[10] metafor: Meta-analysis package for R. The Comprehensive R Archive Network. Accessed June 8, 2023. http://CRAN.R-project.org/package=metafor

[11] Harrell FE. Regression Modeling Strategies: With Applications to Linear Models, Logistic and Ordinal Regression, and Survival Analysis. Springer International Publishing; 2015. doi:10.1007/978-3-319-19425-7

[12] Bodilsen J, Nielsen PB, Søgaard M, . Hospital admission and mortality rates for non-COVID diseases in Denmark during COVID-19 pandemic: nationwide population based cohort study. BMJ. 2021;373(1135):n1135. doi:10.1136/bmj.n1135 34035000 PMC8142604

[13] Razak F, Shin S, Naylor CD, Slutsky AS. Canada’s response to the initial 2 years of the COVID-19 pandemic: a comparison with peer countries. CMAJ. 2022;194(25):E870-E877. doi:10.1503/cmaj.220316 35760433 PMC9332918

[14] Griffith GJ, Morris TT, Tudball MJ, . Collider bias undermines our understanding of COVID-19 disease risk and severity. Nat Commun. 2020;11(1):5749. doi:10.1038/s41467-020-19478-2 33184277 PMC7665028

[15] Huggins A, Husaini M, Wang F, . Care disruption during COVID-19: a national survey of hospital leaders. J Gen Intern Med. 2023;38(5):1232-1238. doi:10.1007/s11606-022-08002-5 36650332 PMC9845025

[16] Health at a glance 2021: OECD indicators. hospital beds and occupancy. Organisation for Economic Co-operation and Development. Accessed May 14, 2023. https://www.oecd-ilibrary.org/sites/e5a80353-en/index.html?itemId=/content/component/e5a80353-en

[17] McAlister FA, Parikh H, Lee DS, Wijeysundera HC. Health care implications of the COVID-19 pandemic for the cardiovascular practitioner. Can J Cardiol. 2023;39(6):716-725. doi:10.1016/j.cjca.2022.11.014 36481398 PMC9721374

[18] Roth GA, Emmons-Bell S, Alger HM, . Trends in patient characteristics and COVID-19 in-hospital mortality in the United States during the COVID-19 pandemic. JAMA Netw Open. 2021;4(5):e218828. doi:10.1001/jamanetworkopen.2021.8828 33938933 PMC8094014

[19] Becker NV, Karmakar M, Tipirneni R, Ayanian JZ. Trends in hospitalizations for ambulatory care-sensitive conditions during the COVID-19 pandemic. JAMA Netw Open. 2022;5(3):e222933. doi:10.1001/jamanetworkopen.2022.2933 35297972 PMC8931555

[20] Cassell K, Zipfel CM, Bansal S, Weinberger DM. Trends in non-COVID-19 hospitalizations prior to and during the COVID-19 pandemic period, United States, 2017-2021. Nat Commun. 2022;13(1):5930. doi:10.1038/s41467-022-33686-y 36209210 PMC9546751

[21] Moynihan R, Sanders S, Michaleff ZA, . Impact of COVID-19 pandemic on utilisation of healthcare services: a systematic review. BMJ Open. 2021;11(3):e045343. doi:10.1136/bmjopen-2020-045343 33727273 PMC7969768

