# Supplementary material for: Outcomes Among Patients Hospitalized With Non–COVID-19 Conditions Before and During the COVID-19 Pandemic in Alberta and Ontario, Canada
Source: JAMA Netw Open. 2023 Jul 12;6(7):e2323035. doi: 10.1001/jamanetworkopen.2023.23035 (PMC10339156; doi:10.1001/jamanetworkopen.2023.23035)
Supplement: Supplement 1. — eTable 1. Frequency of the Medical Indicator Conditions eTable 2. Baseline Characteristics of Patients Hospitalized During the COVID-19 Pandemic, by Surge Stratum eTable 3. Adjusted Risk of 30-Day Mortality and Length of Stay Among Patients Hospitalized During the COVID-19 Pandemic eTable 4. Outcomes for Patients Hospitalized With COVID-19 as a Most Responsible Diagnosis Between April 2020 and September 2021 eFigure. Crude Outcome Rates by Wave and Surge Index, Alberta and Ontario Combined [file jamanetwopen-e2323035-s001.pdf]

Supplemental Online Content

McAlister FA, Chu A, Qiu F, et al; CORONA Collaboration. Outcomes among patients hospitalized with non–COVID-19 conditions before and during the COVID-19 pandemic in Alberta and Ontario, Canada. *JAMA Netw Open*. 2023;6(7):e2323035. doi:10.1001/jamanetworkopen.2023.23035

eTable 1. Frequency of the Medical Indicator Conditions

eTable 2. Baseline Characteristics of Patients Hospitalized During the COVID-19 Pandemic, by Surge Stratum

eTable 3. Adjusted Risk of 30-Day Mortality and Length of Stay Among Patients Hospitalized During the COVID-19 Pandemic

eTable 4. Outcomes for Patients Hospitalized With COVID-19 as a Most Responsible Diagnosis Between April 2020 and September 2021

eFigure. Crude Outcome Rates by Wave and Surge Index, Alberta and Ontario Combined

This supplemental material has been provided by the authors to give readers additional information about their work.

© 2023 McAlister FA et al. *JAMA Network Open.*

eTable 1. Frequency of the Medical Indicator Conditions

Medical condition	Alberta			Ontario		
	Pre-Pandemic (April 2018-September 2019)	Pandemic (April 2020-September 2021)	Admissions with SARS-CoV-2 positivity	Pre-Pandemic (April 2018-September 2019)	Pandemic (April 2020-September 2021)	Admissions with SARS-CoV-2 positivity
Total hospitalizations, n	232,518	215,209	8,837	781,744	709,920	27,778
Patients hospitalized for any of the 5 indicator conditions, n (%)						
Acute coronary syndrome	7552 (3.2%)	7101 (3.3%)	31 (0.4%)	27,945 (3.6%)	26,269 (3.7%)	148 (0.6%)
Chronic obstructive pulmonary disease (COPD)/asthma without COVID specific code U07	6868 (3.0%)	3996 (1.9%)	26 (0.3%)	23,394 (3.0%)	12,960 (1.8%)	42 (0.3%)
Heart failure	5421 (2.3%)	5185 (2.4%)	41 (0.5%)	21,716 (2.8%)	21,132 (3.0%)	145 (0.7%)
Stroke (ischemic or hemorrhagic)	4258 (1.8%)	4200 (2.0%)	53 (0.6%)	21,409 (2.7%)	21,815 (3.1%)	227 (1.0%)
Urinary tract infection/urosepsis	3225 (1.4%)	3027 (1.4%)	25 (0.3%)	10,902 (1.4%)	9,940 (1.4%)	75 (0.8%)

© 2023 McAlister FA et al. *JAMA Network Open.*

eTable 2. Baseline Characteristics of Patients Hospitalized During the COVID-19 Pandemic, by Surge Stratum

Characteristic	Alberta surge stratum						Ontario surge stratum					
	<50th	50th-74th	75th-89th	90th-99th	>99th	P Trend	<50th	50th-74th	75th-89th	90th-99th	>99th	P Trend
Patients, n	2934	9887	5795	4520	308		21,947	30,986	21,905	15,670	1273	
Age, mean (SD)	73.8 (14.5)	70.4 (14.9)	70.2 (15.1)	69.9 (15.0)	70.0 (15.6)	<.001	72.8 (13.8)	72.2 (14.6)	72.0 (14.6)	71.9 (14.6)	72.4 (14.6)	<.001
Male	1508 (51.4)	5537 (56.0)	3332 (57.5)	2532 (56.0)	165 (53.6)	<.001	11,769 (53.6)	16,880 (54.5)	12,006 (54.8)	8,737 (55.8)	701 (55.1)	<.001
Rural residence	1441 (49.1)	1369 (13.8)	836 (14.4)	594 (13.1)	40 (13.0)	<.001	6,470 (29.5)	3,395 (11.0)	1,670 (7.6)	709 (4.5)	19 (1.5)	<.001
Deprivation quintile												
1 (least deprived)	140 (4.8)	1424 (14.4)	824 (14.2)	650 (14.4)	67 (21.8)	<.001	3222 (14.7)	5759 (18.6)	4002 (18.3)	2794 (17.8)	218 (17.1)	0.04
2	342 (11.7)	1395 (14.1)	926 (16.0)	608 (13.5)	41 (13.3)		3978 (18.1)	5716 (18.4)	4084 (18.6)	2920 (18.6)	203 (15.9)	
3	520 (17.7)	1594 (16.1)	955 (16.5)	822 (18.2)	52 (16.9)		4406 (20.1)	5797 (18.7)	4259 (19.4)	2901 (18.5)	284 (22.3)	
4	802 (27.3)	1906 (19.3)	1105 (19.1)	899 (19.9)	54 (17.5)		4742 (21.6)	6172 (19.9)	4354 (19.9)	3303 (21.1)	266 (20.9)	
5 (most deprived)	756 (25.8)	2164 (21.9)	1242 (21.4)	999 (22.1)	56 (18.2)		5092 (23.2)	7157 (23.1)	5022 (22.9)	3640 (23.2)	296 (23.3)	
Unknown	374 (12.7)	1404 (14.2)	743 (12.8)	542 (12.0)	38 (12.3)		507 (2.3)	385 (1.2)	184 (0.8)	112 (0.7)	6 (0.5)	
Long-term care resident	19 (0.6)	211 (2.1)	98 (1.7)	44 (1.0)	8 (2.6)	0.34	672 (3.1)	942 (3.0)	641 (2.9)	398 (2.5)	35 (2.7)	0.004
Non-elective hospitalization in the 12 months prior to index hospitalization	764 (26.0)	1745 (17.6)	391 (6.7)	165 (3.7)	2 (0.6)	<.001	3663 (16.7)	4266 (13.8)	2038 (9.3)	1176 (7.5)	19 (1.5)	<.001
Non-elective hospitalization in the 3 months prior to index hospitalization	189 (6.4)	431 (4.4)	49 (0.8)	2 (0.0)	0 (0.0)	<.001	536 (2.4)	922 (3.0%)	634 (2.9)	*413-417	*1-5	0.67
Unplanned ED visit in the 6 months prior to index hospitalization	699 (23.8)	1535 (15.5)	146 (2.5)	9 (0.2)	1 (0.3)	<.001	7332 (33.4)	9084 (29.3%)	5959 (27.2)	4072 (26.0)	331 (26.0)	<.0001
**Comorbidities from index hospitalization episode and hospitalizations in prior year**												
Coronary artery disease	801 (27.3)	3632 (36.7)	2151 (37.1)	1729 (38.3)	127 (41.2)	<.001	7946 (36.2)	10,860 (35.0)	7993 (36.5)	5974 (38.1)	459 (36.1)	<.001
Heart failure	989 (33.7)	2814 (28.5)	1535 (26.5)	1238 (27.4)	70 (22.7)	<.001	6402 (29.2)	9365 (30.2)	6619 (30.2)	4938 (31.5)	433 (34.0)	<.001
Hypertension	1074 (36.6)	4199 (42.5)	2356 (40.7)	1933 (42.8)	117 (38.0)	0.007	9067 (41.3)	14,412 (46.5)	10,660 (48.7)	8192 (52.3)	726 (57.0)	<.001

© 2023 McAlister FA et al. *JAMA Network Open.*

Peripheral arterial disease	59 (2.0)	100 (1.0)	44 (0.8)	47 (1.0)	4 (1.3)	<.001	424 (1.9)	589 (1.9)	393 (1.8)	292 (1.9)	28 (2.2)	0.59
Ventricular arrythmias	24 (0.8)	143 (1.4)	78 (1.3)	66 (1.5)	4 (1.3)	0.14	338 (1.5)	537 (1.7)	391 (1.8)	273 (1.7)	14 (1.1)	0.26
Atrial fibrillation or flutter	395 (13.5)	1304 (13.2)	727 (12.5)	621 (13.7)	31 (10.1)	0.73	3598 (16.4)	5555 (17.9)	3833 (17.5)	2775 (17.7)	229 (18.0)	0.004
Cancer	130 (4.4)	303 (3.1)	116 (2.0)	88 (1.9)	6 (1.9)	<.001	960 (4.4)	1415 (4.6)	985 (4.5)	669 (4.3)	54 (4.2)	0.58
Chronic pulmonary disease or asthma	984 (33.5)	2044 (20.7)	1044 (18.0)	834 (18.5)	53 (17.2)	<.001	4953 (22.6)	5913 (19.1)	3690 (16.8)	2355 (15.0)	201 (15.8)	<.001
Dementia	165 (5.6)	420 (4.2)	204 (3.5)	140 (3.1)	8 (2.6)	<.001	1064 (4.8)	1629 (5.3)	1048 (4.8)	837 (5.3)	68 (5.3)	0.23
Diabetes	942 (32.1)	3094 (31.3)	1669 (28.8)	1414 (31.3)	93 (30.2)	0.06	7009 (31.9)	9681 (31.2)	6889 (31.4)	4983 (31.8)	455 (35.7)	0.47
Liver disease	29 (1.0)	116 (1.2)	48 (0.8)	52 (1.2)	2 (0.6)	0.60	267 (1.2)	396 (1.3)	252 (1.2)	208 (1.3)	12 (0.9)	0.94
Other lung disease (non-COPD/asthma)	94 (3.2)	338 (3.4)	160 (2.8)	134 (3.0)	4 (1.3)	0.040	505 (2.3)	873 (2.8)	567 (2.6)	439 (2.8)	32 (2.5)	0.031
Peptic ulcer disease	21 (0.7)	101 (1.0)	30 (0.5)	32 (0.7)	3 (1.0)	0.07	126 (0.6)	184 (0.6)	112 (0.5)	91 (0.6)	12 (0.9)	0.90
Renal disease	222 (7.6)	525 (5.3)	227 (3.9)	233 (5.2)	8 (2.6)	<.001	1114 (5.1)	1801 (5.8)	1200 (5.5)	873 (5.6)	71 (5.6)	0.14
Cerebrovascular disease (includes stroke and TIA)	360 (12.3)	1869 (18.9)	1225 (21.1)	886 (19.6)	50 (16.2)	<.001	4557 (20.8)	7947 (25.6)	5758 (26.3)	4146 (26.5)	332 (26.1)	<.001
**Characteristics of index hospitalization**												
Schneeweiss Charlson score, mean (SD)	2.89 (2.20)	2.57 (1.97)	2.34 (1.74)	2.46 (1.81)	2.17 (1.76)	<.001	0.93 (1.67)	0.82 (1.60)	0.69 (1.46)	0.65 (1.45)	0.52 (1.21)	<.001
Number of acute hospital beds												
<100	2343 (79.9)	1283 (13.0)	774 (13.4)	381 (8.4)	49 (15.9)	<.001	8062 (36.7)	4498 (14.5)	2606 (11.9)	1138 (7.3)	127 (10.0)	<.001
100-199	403 (13.7)	2753 (27.8)	1621 (28.0)	1866 (41.3)	259 (84.1)		5731 (26.1)	6495 (21.0)	5109 (23.3)	3542 (22.6)	429 (33.7)	
200-299	0 (0.0)	469 (4.7)	407 (7.0)	328 (7.3)	0 (0.0)		2229 (10.2)	5527 (17.8)	4114 (18.8)	3705 (23.6)	366 (28.8)	
300-399	0 (0.0)	2286 (23.1)	650 (11.2)	806 (17.8)	0 (0.0)		4429 (20.2)	7284 (23.5)	4379 (20.0)	2856 (18.2)	105 (8.2)	
400-499	188 (6.4)	1331 (13.5)	524 (9.0)	952 (21.1)	0 (0.0)		322 (1.5)	2078 (6.7)	1547 (7.1)	1531 (9.8)	146 (11.5)	
≥500	0 (0.0)	1765 (17.9)	1819 (31.4)	187 (4.1)	0 (0.0)		1174 (5.3)	5104 (16.5)	4150 (18.9)	2898 (18.5)	100 (7.9)	
Admission to teaching hospital	281 (9.6)	3213 (32.5)	2427 (41.9)	1248 (27.6)	18 (5.8)	<.001	6278 (28.6)	11,760 (38.0)	6019 (27.5)	3351 (21.4)	67 (5.3)	<.001
Admission to ICU	148 (5.0)	676 (6.8)	382 (6.6)	440 (9.7)	16 (5.2)	<.001	5927 (27.0%)	9188 (29.7%)	6681 (30.5%)	4801 (30.6%)	265 (20.8%)	<.001
Received mechanical ventilation	82 (2.8)	481 (4.9)	331 (5.7)	264 (5.8)	21 (6.8)	<.001	1507 (6.9%)	2422 (7.8%)	1719 (7.8%)	1207 (7.7%)	86 (6.8%)	<.001
Total ICU hours, median (Q1-Q3)	59 (31, 101)	58 (32, 98)	58 (34, 102)	47 (25, 79)	24 (13, 50)	<.001	63 (35-112)	61 (33-117)	60 (33-110)	52 (28-96)	55 (36-95)	<.001
Episode length of stay, days, mean (SD)	10.9 (21.2)	9.9 (18.8)	9.9 (16.7)	9.5 (16.7)	6.9 (7.6)	<.001	8.6 (15.2)	8.9 (15.0)	9.0 (16.1)	9.0 (15.2)	8.6 (13.1)	0.02

© 2023 McAlister FA et al. *JAMA Network Open.*

Discharge disposition												
Long-term care/skilled nursing facility	382 (13.0)	669 (6.8)	386 (6.7)	285 (6.3)	25 (8.1)	<.001	3732 (17.0)	6084 (19.6)	4167 (19.0)	3036 (19.4)	242 (19.0)	<.001
Home with home care	413 (14.1)	1641 (16.6)	933 (16.1)	586 (13.0)	44 (14.3)		5832 (26.6)	8520 (27.5)	5931 (27.1)	4425 (28.2)	400 (31.4)	
Home without home care	1921 (65.5)	6867 (69.5)	4075 (70.3)	3303 (73.1)	213 (69.2)		10,817 (49.3)	14,125 (45.6)	10,211 (46.6)	7,080 (45.2)	541 (42.5)	
Death	218 (7.4)	710 (7.2)	401 (6.9)	346 (7.7)	26 (8.4)		1566 (7.1)	2257 (7.3)	1596 (7.3)	1129 (7.2)	90 (7.1)	
Positive SARS-CoV-2 test result	3 (0.1)	31 (0.3)	51 (0.9)	85 (1.9)	5 (1.6)	<.001	17 (0.1)	112 (0.4)	174 (0.8)	290 (1.9)	43 (3.4)	<.001
Surge index, mean (SD)	0.00 (0.01)	0.62 (0.38)	2.80 (1.07)	8.26 (2.93)	20.31 (4.71)	<.001	0.04 (0.06)	0.70 (0.36)	2.65 (0.91)	8.05 (2.69)	21.80 (7.40)	<.001

Abbreviations: COPD, chronic obstructive pulmonary disease; ED, emergency department; ICU, intensive care unit; SD, standard deviation; TIA, transient ischemic attack.

© 2023 McAlister FA et al. *JAMA Network Open.*

eTable 3. Adjusted Risk of 30-Day Mortality and Length of Stay Among Patients Hospitalized During the COVID-19 Pandemic

Condition	Surge index category	Alberta	Ontario	Pooled	Alberta	Ontario	Pooled
		30-day mortality, adjusted Odds Ratio (95% CI)			Length of stay, adjusted Odds Ratio (95% CI)		
Heart failure and negative for SARS-CoV2 infection	<50%	Ref	Ref	Ref	Ref	Ref	Ref
	50%-74%	1.34 (0.97, 1.85)	0.91 (0.79, 1.04)	1.07 (0.74, 1.56)	1.04 (0.95, 1.13)	0.99 (0.96, 1.02)	1 (0.96, 1.03)
	75%-89%	1.13 (0.78, 1.63)	0.99 (0.86, 1.15)	1.01 (0.88, 1.15)	1.01 (0.92, 1.11)	1.01 (0.97, 1.04)	1.01 (0.98, 1.04)
	90%-99%	1.33 (0.91, 1.95)	1.09 (0.93, 1.27)	1.12 (0.97, 1.3)	0.98 (0.89, 1.08)	0.98 (0.95, 1.02)	0.98 (0.95, 1.01)
	>99%	1.64 (0.66, 4.05)	1.18 (0.81, 1.72)	1.24 (0.87, 1.75)	0.88 (0.69, 1.11)	1.03 (0.94, 1.12)	0.99 (0.87, 1.13)
Acute coronary syndrome and negative for SARS-CoV2 infection	<50%	Ref	Ref	Ref	Ref	Ref	Ref
	50%-74%	2.19 (1.30, 3.69)	1.06 (0.92, 1.23)	1.46 (0.72, 2.95)	0.94 (0.88, 1.01)	1.02 (1.00, 1.04)	0.99 (0.91, 1.07)
	75%-89%	1.92 (1.10, 3.34)	1.05 (0.89, 1.23)	1.34 (0.75, 2.38)	0.98 (0.91, 1.06)	0.99 (0.97, 1.02)	0.99 (0.97, 1.01)
	90%-99%	2.01 (1.15, 3.52)	1.04 (0.87, 1.24)	1.37 (0.72, 2.58)	0.97 (0.90, 1.05)	0.96 (0.94, 0.99)	0.96 (0.94, 0.98)
	>99%	2.47 (0.91, 6.71)	0.95 (0.57, 1.59)	1.39 (0.55, 3.46)	0.84 (0.73, 0.97)	0.92 (0.86, 0.99)	0.9 (0.83, 0.97)
Stroke and negative for SARS-CoV2 infection	<50%	Ref	Ref	Ref	Ref	Ref	Ref
	50%-74%	0.78 (0.54, 1.13)	0.98 (0.87, 1.11)	0.94 (0.78, 1.12)	1.05 (0.91, 1.21)	0.98 (0.95, 1.01)	0.98 (0.95, 1.01)
	75%-89%	0.93 (0.63, 1.37)	1.03 (0.90, 1.18)	1.02 (0.9, 1.16)	1.05 (0.91, 1.22)	1.01 (0.98, 1.05)	1.01 (0.98, 1.05)
	90%-99%	0.84 (0.56, 1.25)	1.01 (0.87, 1.17)	0.99 (0.86, 1.14)	0.95 (0.82, 1.11)	0.96 (0.92, 1.00)	0.96 (0.92, 1)
	>99%	2.30 (1.02, 5.16)	1.18 (0.81, 1.70)	1.49 (0.8, 2.78)	0.66 (0.47, 0.92)	0.92 (0.83, 1.02)	0.81 (0.59, 1.11)
Chronic obstructive pulmonary disease/asthma and negative for SARS-CoV2 infection	<50%	Ref	Ref	Ref	Ref	Ref	Ref
	50%-74%	0.88 (0.60, 1.31)	1.00 (0.84, 1.17)	0.98 (0.84, 1.14)	1.00 (0.91, 1.09)	1.00 (0.97, 1.04)	1 (0.97, 1.03)
	75%-89%	0.95 (0.61, 1.49)	1.03 (0.85, 1.24)	1.02 (0.86, 1.21)	1.14 (1.03, 1.26)	0.99 (0.95, 1.03)	1.05 (0.92, 1.21)
	90%-99%	1.03 (0.64, 1.66)	1.03 (0.83, 1.29)	1.03 (0.84, 1.26)	1.04 (0.94, 1.16)	1.03 (0.98, 1.08)	1.03 (0.99, 1.08)
	>99%	2.50 (0.95, 6.61)	1.32 (0.74, 2.37)	1.61 (0.9, 2.86)	0.87 (0.68, 1.12)	0.99 (0.87, 1.13)	0.96 (0.86, 1.08)
Urinary tract infection/urosepsis and negative for	<50%	Ref	Ref	Ref	Ref	Ref	Ref
	50%-74%	0.77 (0.43, 1.37)	0.92 (0.70, 1.20)	0.89 (0.70, 1.14)	1.05 (0.94, 1.17)	1.02 (0.98, 1.07)	1.02 (0.98, 1.07)
	75%-89%	0.75 (0.39, 1.42)	0.89 (0.65, 1.21)	0.86 (0.65, 1.14)	1.03 (0.91, 1.16)	1.07 (1.02, 1.13)	1.06 (1.01, 1.12)
	90%-99%	0.88 (0.44, 1.75)	0.91 (0.64, 1.30)	0.90 (0.66, 1.24)	0.96 (0.84, 1.09)	1.06 (1.00, 1.12)	1.03 (0.94, 1.12)

© 2023 McAlister FA et al. *JAMA Network Open.*

SARS-CoV2 infection	>99%	0.34 (0.04, 2.87)	1.10 (0.42, 2.89)	0.90 (0.37, 2.17)	1.24 (0.93, 1.65)	1.22 (1.05, 1.43)	1.22 (1.07, 1.4)
COVID-19 most responsible diagnosis	<75%	Ref	Ref	Ref	Ref	Ref	Ref
	75%-89%	1.26 (0.92, 1.73)	1.23 (1.05, 1.44)	1.24 (1.08, 1.42)	0.95 (0.88, 1.03)	1.30 (1.12, 1.51)	1.10 (0.81, 1.50)
	90%-99%	1.47 (1.09, 1.98)	1.34 (1.16, 1.55)	1.36 (1.20, 1.56)	0.90 (0.84, 0.97)	1.35 (1.17, 1.56)	1.10 (0.74, 1.63)
	>99%	2.29 (1.52, 3.44)	1.55 (1.29, 1.85)	1.80 (1.24, 2.61)	0.93 (0.84, 1.03)	1.42 (1.19, 1.69)	1.14 (0.76, 1.72)

Adjusted for age, sex, coronary artery disease, heart failure, hypertension, peripheral artery disease, ventricular arrythmias, atrial fibrillation or flutter, cancer, chronic pulmonary disease, other lung disease, dementia, diabetes, liver disease, peptic ulcer disease, renal disease, cerebrovascular disease (stroke and transient ischemic attack), Charlson comorbidity index score, type of hospital (teaching vs non-teaching), patient residence at time of admission (rural vs. urban, long-term care facility vs. community dwelling), socioeconomic status (deprivation index), number of non-elective hospitalizations in the 12 months prior to index hospitalization, and number of emergency department visits in the six months.

© 2023 McAlister FA et al. *JAMA Network Open.*

eTable 4. Outcomes for Patients Hospitalized With COVID-19 as a Most Responsible Diagnosis Between April 2020 and September 2021*

	Alberta				Ontario			
	Patients	30 day mortality, n (%)	In-hospital mortality, n (%)	Length of stay, median days (Q1-Q3)	Patients	30 day mortality, n (%)	In-hospital mortality, n (%)	Length of stay, median days (Q1-Q3)
Pandemic (Apr 2020 to Sep 2021)	7409	1081 (14.59)	1030 (13.90)	7 (4-14)	23,803	3926 (16.5)	4253 (17.9)	8 (4-16)
Surge index percentile <50th	≤5	Suppressed	Suppressed	Suppressed	105	6 (5.7)	8 (7.6)	8 (4-14)
50th-74th	687	87 (12.7)	83 (12.1)	8 (4-14)	2229	327 (14.7)	343 (15.4)	8 (4-16)
75th-89th	1819	256 (14.1)	239 (13.1)	7 (4-14)	5395	907 (16.8)	988 (18.3)	8 (4-17)
90th-99th	4261	652 (15.3)	625 (14.7)	7 (4-14)	12,561	2170 (17.3)	2354 (18.7)	8 (4-16)
>99th	639	86 (13.5)	83 (13.0)	7 (4-13)	3513	516 (14.7)	560 (15.9)	8 (4-15)

* For privacy concerns, results and counts have been suppressed where cells contain 5 or less individuals

© 2023 McAlister FA et al. *JAMA Network Open.*

eFigure. Crude Outcome Rates by Wave and Surge Index, Alberta and Ontario Combined

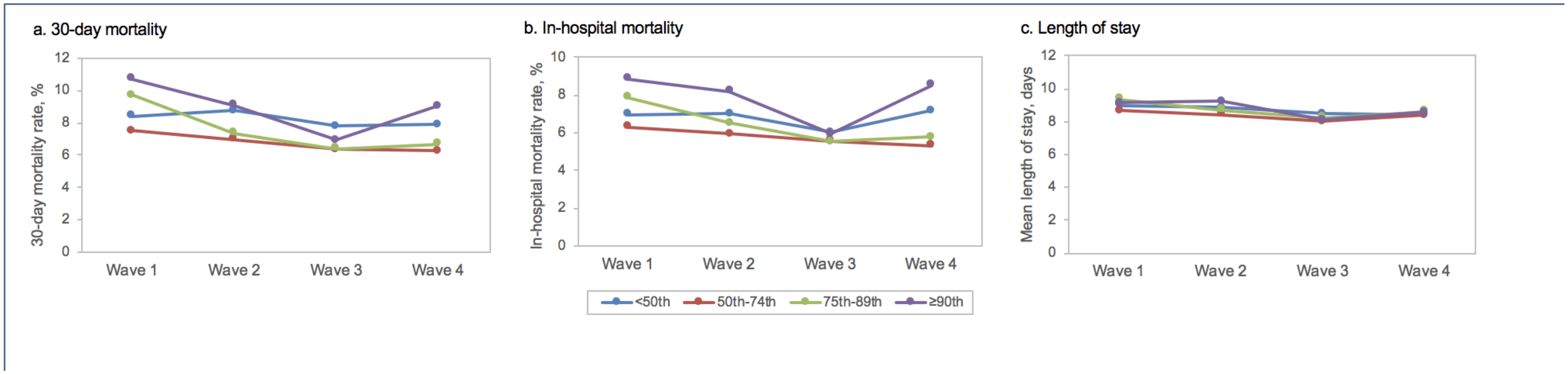

© 2023 McAlister FA et al. *JAMA Network Open.*
