# Supplementary material for: Outcomes Among Patients Hospitalized With Non–COVID-19 Conditions Before and During the COVID-19 Pandemic in Alberta and Ontario, Canada
Source: JAMA Netw Open. 2023 Jul 12;6(7):e2323035. doi: 10.1001/jamanetworkopen.2023.23035 (PMC10339156; doi:10.1001/jamanetworkopen.2023.23035)
Supplement: Supplement 2. — Nonauthor Collaborators [file jamanetwopen-e2323035-s002.pdf]

Supplemental Online Content: Nonauthor Collaborators
*First name, last name, and suffix (if applicable) are required and will appear in PubMed.

***Group Name(s): CORONA Collaboration**

***First Name and Middle Initial(s)**	***Last Name**	***Suffix (eg, Jr, III)**	Academic Degrees	Institution	Location (city, state/province, country)	Role or Contribution, eg, chair, principal investigator	Group (if more than 1 Group listed in the byline) and/or Subgroup (eg, Steering Committee)
Husam	Abdel-Qadir		MD, PhD, FRCP(	Women's College Hospital	Toronto, ON, Canada	Co-Investigator	
Peter C	Austin		PhD	ICES	Toronto, ON, Canada	Co-Investigator	
Kevin	Bainey		MD	University of Alberta	Edmonton, AB, Canada	Co-Investigator	
Jeff	Bakal		PhD	Alberta Health Services		Co-Investigator	
Charles	de Mestral		MD, PhD	Unity Health Toronto	Toronto, ON, Canada	Co-Investigator	
Justin	Ezekowitz		MBBCh, MSc	University of Alberta	Edmonton, AB, Canada	Co-Investigator	
Shaun	Goodman		MD, MSc	St. Michael's Hospital	Toronto, ON, Canada	Co-Investigator	
Russ	Greiner		PhD	University of Alberta	Edmonton, AB, Canada	Co-Investigator	
Andrew	Ha		MD, MSc	University Health Network	Toronto, ON, Canada	Co-Investigator	
Cynthia	Jackevicius		PharmD, MSc, BScPhm	ICES	Toronto, ON, Canada	Co-Investigator	
Sunil V	Kalmady		PhD, MSc	University of Alberta	Edmonton, AB, Canada	Collaborator	
Moira K	Kapral		MD, MSc, FRCPC	University of Toronto	Toronto, ON, Canada	Co-Investigator	
Padma	Kaul		PhD	University of Alberta	Edmonton, ON, Canada	Co-Investigator	
Dennis T	Ko		MD	ICES	Toronto, ON, Canada	Co-Investigator	
Jeff	Kwong		MD, MSc, CCFP, FRCPC	ICES	Toronto, ON, Canada	Co-Investigator	
Douglas S	Lee		MD, PhD, FRCPC	University Health Network	Toronto, ON, Canada	Principal Investigator	
Peter	Liu		MSc, MD, FRCPC	University of Ottawa Heart Institute	Ottawa, ON, Canada	Co-Investigator	
Finlay A	McAlister		MC, MSc	University of Alberta	Edmonton, AB, Canada	Principal Investigator	
Paula A	Rochon		MD, MPH, FRCPC	Women's College Hospital	Toronto, ON, Canada	Co-Investigator	
Idan	Roifman		MD, MSc	Sunnybrook Research Institute	Toronto, ON, Canada	Co-Investigator	
Heather J	Ross		MD, MHSc, FRCPC FACC, FCCS	University Health Network	Toronto, ON, Canada	Co-Investigator	
Roopinder	Sandhu		MD, MPH	University of Alberta	Edmonton, AB, Canada	Co-Investigator	

Supplemental Online Content: Nonauthor Collaborators

*First name, last name, and suffix (if applicable) are required and will appear in PubMed.

***First Name and Middle Initial(s)**	***Last Name**	***Suffix (eg, Jr, III)**	Academic Degrees	Institution	Location (city, state/province, country)	Role or Contribution, eg, chair, principal investigator	Group (if more than 1 Group listed in the byline) and/or Subgroup (eg, Steering Committee)
Michael J	Schull		MD, MSc, FRCPC	ICES	Toronto, ON, Canada	Co-Investigator	
Louise	Sun		MD, SM, FRCPC	University of Ottawa Heart Institute	Ottawa, ON, Canada	Co-Investigator	
Jacob A	Udell		MD, MPH, FRCPC	Women's College Hospital	Toronto, ON, Canada	Co-Investigator	
Sean	van Diepen		MD, MSc	University of Alberta	Edmonton, AB, Canada	Co-Investigator	
Bo	Wang		PhD	University Health Network	Toronto, ON, Canada	Co-Investigator	
Robert	Welsh		MD	University of Alberta	Edmonton, AB, Canada	Co-Investigator	
Harindra	Wijeysundera		MD, PhD	Sunnybrook Research Institute	Toronto, ON, Canada	Co-Investigator	
Amy	Yu		MD, MSc	Sunnybrook Research Institute	Toronto, ON, Canada	Co-Investigator	
