# Supplementary material for: Outcomes Among Patients Hospitalized With Non–COVID-19 Conditions Before and During the COVID-19 Pandemic in Alberta and Ontario, Canada
Source: JAMA Netw Open. 2023 Jul 12;6(7):e2323035. doi: 10.1001/jamanetworkopen.2023.23035 (PMC10339156; doi:10.1001/jamanetworkopen.2023.23035)
Supplement: Supplement 3. — Data Sharing Statement [file jamanetwopen-e2323035-s003.pdf]

Data Sharing Statement

McAlister. Outcomes Among Patients Hospitalized With Non–COVID-19 Conditions Before and During the COVID-19 Pandemic in Alberta and Ontario, Canada. *JAMA Netw Open*. Published July 12, 2023. doi:10.1001/jamanetworkopen.2023.23035

Data
Data available: No

Additional Information
Explanation for why data not available: To comply with each province's Health Information Protection Act and in order to minimize the possibility of unintentionally sharing information that can be used to re-identify private information, the dataset cannot be made publicly available.
